# Brain-derived neurotrophic factor exerts neuroprotective actions against amyloid β-induced apoptosis in neuroblastoma cells

**DOI:** 10.3892/etm.2014.2033

**Published:** 2014-10-17

**Authors:** JIN HEE KIM

**Affiliations:** Department of Neurosurgery, College of Medicine, Korea University, Seoul 136-701, Republic of Korea

**Keywords:** β-amyloid peptide, brain-derived neurotrophic factor, Akt, glycogen synthase kinase 3β, neuroblastoma cell

## Abstract

Alzheimer’s disease (AD) brains demonstrate decreased levels of brain-derived neurotrophic factor (BDNF) and increased levels of β-amyloid peptide (Aβ), which is neurotoxic. The present study assessed the impact of BDNF on the toxic effects of Aβ_25–35_-induced apoptosis and the effects on BDNF-mediated signaling using the MTT assay, western blotting and reverse transcription quantitative polymerase chain reaction. Aβ_25–35_ was found to induce an apoptosis, dose-dependent effect on SH-SY5Y neuroblastoma cells, which peaked at a concentration of 20 μM after 24 h. A combination of Aβ_25–35_ and BDNF treatment increased the levels of Akt and decreased the level of glycogen synthase kinase-3β (GSK3β) in SH-SY5Y neuroblastoma cells. These findings indicated that BDNF administration exerted a neuroprotective effect against the toxicity of the Aβ_25–35_-induced apoptosis in these cells, which was accompanied by phosphoinositide 3-kinase/Akt activation and GSK3β phosphorylation. The mechanisms and signaling pathways underlying neuronal degeneration induced by the Aβ peptide remain to be further elucidated.

## Introduction

Alzheimer’s disease (AD) is a neurodegenerative disorder of the human brain and is associated with loss of memory and cognitive abilities (1). AD is characterized by amyloid plaques, neurofibrillary tangles and neuronal loss (2). In AD brains, there is also an abundance of two abnormal structures: Senile plaques composed of β-amyloid peptide (Aβ), that are deposited outside neuronal bodies, and neurofibrillary tangles, which are aggregates of hyperphosphorylated tau proteins that bind to microtubules within the neurons (3). Synaptic dysfunction in AD may be caused by accumulation of aggregated amyloid peptides (3). Aβ, a 40–42 amino acid peptide fragment of the Aβ precursor, has been shown to be a key pathological feature in the formation of senile plaques (4). Aβ peptides induce cell death, decrease survival rate, and increase inflammation, oxidative stress and neurotoxicity in *in vitro* models used to study AD (5,6).

Brain-derived neurotrophic factor (BDNF) is an important neurotrophin that has been extensively studied and that may play a role in the pathology of AD. BDNF is involved in the structural and functional plasticity of the brain (7), protects neurons in the brain against insults (8) and plays a role in neural development and maintenance of the central and peripheral neurons (9). Another important intracellular regulatory protein is glycogen synthase kinase-3β (GSK3β). This protein is phoshorylated by growth factor-stimulated signaling pathways (10). GSK3β is a protein kinase that also has regulatory effects on neuronal survival and plasticity. Previously, a study indicated that GSK3β may play a part in AD and that its deregulation may account for a number of the pathological hallmarks of AD (11).

In the present study, the impact of BDNF on the toxic effects of the Aβ-induced apoptosis was examined via the phosphoinositide 3-kinase (PI3K)/Akt signaling pathway in SH-SY5Y neuroblastoma cells.

## Materials and methods

### Cell culture

Human SH-SY5Y neuroblastoma cells were maintained in Dulbecco’s modified Eagles’s medium (DMEM) and F-12 (Gibco-BRL, Gaithersburg, MD, USA) supplemented with 10% fetal bovine serum (FBS; HyClone, Logan, UT, USA) in a humidified atmosphere of 5% CO_2_ and 95% air at 37°C. The media was replaced every two days. Prior to the experiments, the SH-SY5Y cells were plated in 96-well plates at a density of ~1.5×10^4^ cells per well (for MTT) and in six-well plates at 8×10^6^ cells per well (all other assays). For the experiments, the cells were incubated with agents for 24 h at 37°C. For a single experiment, each treatment was performed in triplicate.

### Reagents

Aβ_25–35_ and scrambled peptides were purchased from Bachem (Weil am Rhein, Germany). BDNF and LY294002 were purchased from Sigma-Aldrich (St Louis, MO, USA). Antibodies against phosphorylated-(p-)Akt (product no. 9271), Akt (product no. 9272), p-GSK3β (product no. 9336) and GSK3β (product no. 9315) were from Cell Signaling Technology, Inc. (Danvers, MA, USA). The antibody against BNDF (product no. 546) was from Santa Cruz Biotechnology, Inc. (Santa Cruz, CA, USA) and the antibody against Actin (product no. 3280) was from Abcam (Cambridge, MA, USA).

### Cell proliferation and MTT assay

A cell survival analysis was performed according to the MTT (Cell Titer 96 Aqueous Cell Proliferation Assay kit; Promega, Madison, WI, USA) assay method. Briefly, the cells were cultured with Aβ (0–20 μM) or BDNF (0–30 ng/ml), and 10 μl of 4 mg/ml MTT solution was added to each well of the 96-well plate. The cells were subsequently incubated for 4 h in the dark. The absorbance was measured in a microplate reader at 490 nm, and the results were expressed as a percentage of the control.

### Evaluation of apoptotic cells by Annexin-V-FITC

Apoptosis was induced by incubating cells with culture medium containing Aβ (20 μM), BDNF (10 ng/ml) and LY294002 (20 μM). The cells were stained with Annexin-V-FITC according to the manufacturer’s instructions (Molecular Probes, Eugene, OR, USA). Approximately 1×10^5^ cells were harvested and washed with phosphate-buffered saline. The cells were resuspended in 100 μl of Annexin-V binding buffer (10 mM 4-(2-hydroxyethyl)-1-piperazineethanesulphonic acid, 140 mM NaCl and 2.5 mM CaCl2; pH 7.4), incubated with 5 μl of Annexin-V-FITC for 15 min at room temperature and counterstained with propidium iodide (final concentration, 1 μg/ml). Following the incubation period, the cells were diluted with 190 μl of Annexin-V binding buffer. The cells were analyzed by flow cytometry using a Becton-Dickinson FACScan flow cytometer with Cell Quest software (Becton-Dickinson, Mountain View, CA, USA).

### Western blotting

The cells were washed in fresh phosphate-buffered saline, homogenized in lysis buffer and centrifuged. Protein concentration was determined by the Bradford assay. The purified proteins were separated by polyacrylamide gel electrophoresis (SDS-PAGE) and the resolved proteins were transferred to a nitrocellulose membrane. Each membrane was incubated overnight with a 1:1000 dilution of the primary antibody at 4°C. The membranes were treated with a 1:1000 dilution of peroxidase-conjugated secondary anti-rabbit or anti-mouse antibodies for 2 h. The proteins were detected using the enhanced chemiluminescence western blotting method (GE Healthcare, Piscataway, NJ, USA). Densitometric quantification of the bands was performed using ImageJ software version 1.29× (National Institutes of Health, Bethesda, MD, USA) (12).

### Reverse transcription quantitative polymerase chain reaction (RT-qPCR) analysis

Total RNA was extracted from the cells following the Promega Total RNA Isolation System manual. RT-qPCR was performed on an ABI Prism 7500 Sequence Detection System (Applied Biosystems, Inc., Foster City, CA, USA), following the manufacturer’s instructions, with SYBR Green (Toyobo Corp., Osaka, Japan) used as a double-stranded DNA specific fluorescent dye. The specific primers were as follows: *GSK3β* forward, 5′-ATCCTTATCCCTCCTCACGC-3′; and reverse, 5′-GTTATTGGTCTGTCCACGGTCT-3′; *Akt* forward, 5′-AGGCATCCCTTCCTTACAGC-3′; and reverse, 5′-CAGCCCGAAGTCCGTTATCT-3′; and *β-actin* forward, F-5′-CGTTGACATCCGTAAAGACCTC-3′; and reverse, 5′-TAGGAGCCAGGGCAGTAATCT-3′.

### Statistical analysis

Data were obtained from three separate cultures and expressed as the mean ± standard error of the mean. Statistical comparison was determined by an analysis of variance test with the Student’s t-test as the post hoc test. P<0.05 was considered to indicate a statistically significant difference.

## Results

### Effect of increasing concentrations of Aβ_25–35_ on cell viability

**Figure 1:**
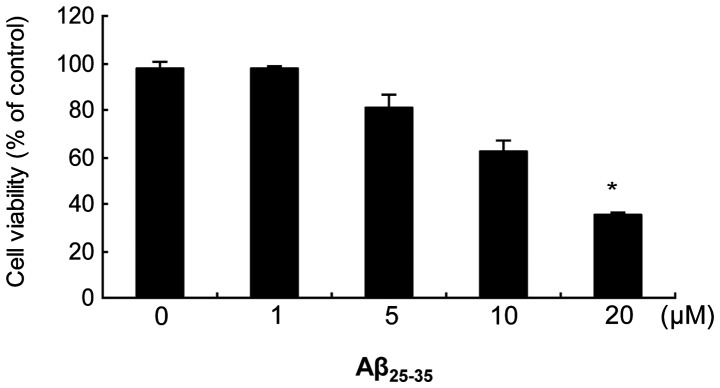
Dose-dependent neurotoxicity of Aβ_25–35_ peptides in human SH-SY5Y neuroblastoma cells. Increasing concentrations of Aβ_25–35_ were added to the culture medium of cells, and the toxicity was estimated after 24 h using the MTT assay. Values represent the mean ± standard error of the mean for three different cultures, with n=3 dishes/culture for each concentration. ^*^P<0.05 compared with the non-treated groups. Aβ, β-amyloid.

The MTT assay was used to determine Aβ_25–35_-induced toxicity (Fig. 1). A 24 h exposure to Aβ_25–35_ induced a toxic, dose-dependent effect on SH-SY5Y cells, with a maximal effect of ~40% noted at a concentration of 20 μM. Thus, 20 μM was used as the concentration for Aβ_25–35_ in all further experiments. The scrambled peptide did not affect cell survival in comparison with the vehicle-treated control group: 100±3% at 20 μM in the Aβ_25–35_ group versus 98±2% in the control group.

### Effect of increasing BDNF concentrations on Aβ_25–35_-treated cells

**Figure 2:**
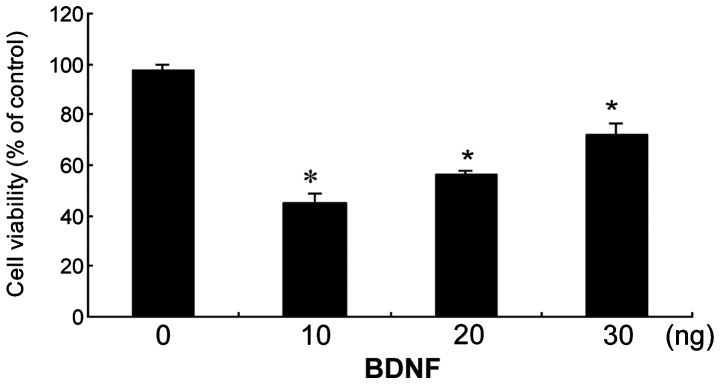
Neuroprotective effect of BDNF on SH-SY5Y neuroblastoma cells exposed to Aβ_25–35_ (20 μM). Aβ_25–35_ (20 μM) was added to cells in the presence of increasing concentrations of BDNF in the culture medium for 24 h, after which toxicity was estimated using the MTT assay. Values represent the mean ± standard error of the mean for three different cultures, with n=3 dishes/culture for each concentration. ^*^P<0.05 compared with groups without BDNF treatment. Aβ, β-amyloid; BDNF, brain-derived neurotrophic factor.

**Figure 3:**
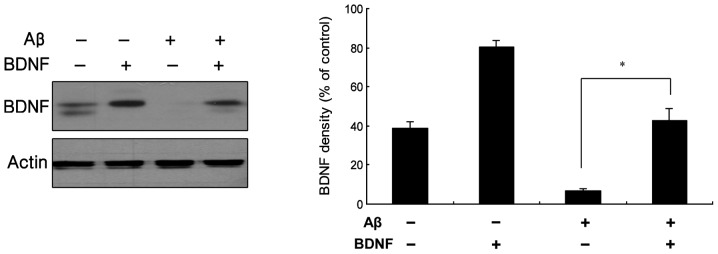
Neuroprotective effect of BDNF on SH-SY5Y neuroblastoma cells exposed to Aβ_25–35_ (20 μM). Aβ_25–35_ (20 μM) was added to cells in the presence of BDNF (10 ng/ml) in medium for 24 h, after which toxicity was estimated by western blotting. Results showed that BDNF levels increased when cells were treated with Aβ_25–35_ (20 μM) combined with BDNF (10 ng/ml). Aβ, β-amyloid; BDNF, brain-derived neurotrophic factor.

The ability of BDNF to protect SH-SY5Y cells against Aβ_25–35_-induced toxicity using the MTT assay was examined (Fig. 2). BDNF was able to protect SH-SY5Y cells from 20 μM Aβ_25–35_-induced toxicity. This protective effect of BDNF was dose-dependent, and the effect was significant ≥10 ng/ml. In Fig. 3, the results from a western blot analysis are shown, indicating that BDNF levels increased following exposure of the cells to treatment with a combination of 20 μM Aβ_25–35_ and 10 ng/ml BDNF.

### BDNF reduces the apoptosis in SH-SY5Y cells

**Figure 4:**
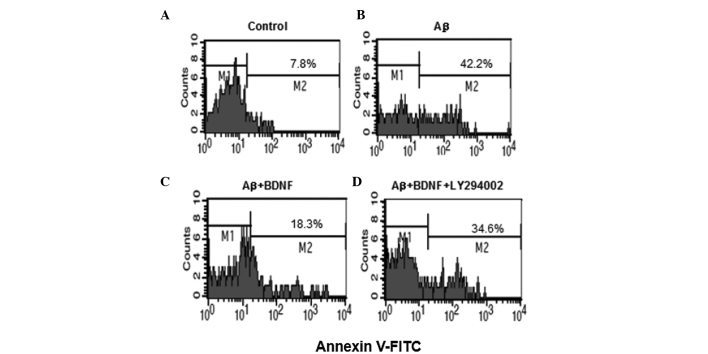
BDNF attenuates Aβ_25–35_-induced apoptosis and inhibition of SH-SY5Y neuroblastoma cells. (A) Flow cytometry assay was performed to visualize the extent of programmed cell death in the control group. (B) Apoptosis of cells that were administered Aβ_25–35_ (20 μM). (C) Apoptosis of cells that were administered Aβ_25–35_ (20 μM) combined with BDNF (10 ng/ml). (D) Apoptosis of cells that were administered Aβ_25–35_ (20 μM) combined with BDNF (10 ng/ml) and LY294002 (20 μM). M2 represents the population of cells with high annexin V binding. Aβ, β-amyloid; BDNF, brain-derived neurotrophic factor.

To examine whether β_25–35_-induced cell death is apoptotic-like, the flow cytometry assay was performed (Fig. 4). In the control, apoptotic cells comprised 7.8% of the total number of cells. Following exposure to 20 μM Aβ_25–35_ for 24 h, the number of apoptotic cells increased to 42.2%. This increase was prevented by addition of BDNF (10 ng/ml). To further investigate, LY294002 (20 μM), the inhibitor of Akt, was used. The apoptosis of cells increased to 34.6% following the use of LY294002 (20 μM).

### BDNF-mediated signal transduction in SH-SY5Y cells

**Figure 5:**
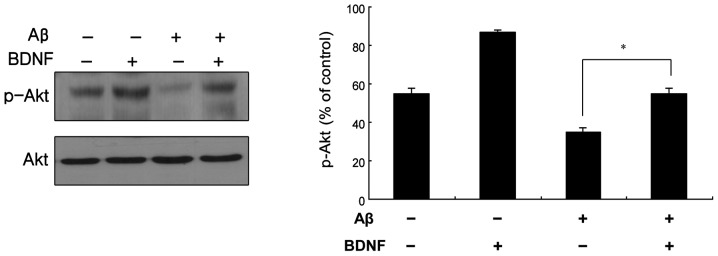
BDNF-mediated activation of Akt in SH-SY5Y neuroblastoma cells. Representative western blots showing protein levels of phosphorylated- and total Akt in SH-SY5Y cells. The western blots presented are representative of three independent experiments with similar results. ^*^P<0.05 vs. the group treated only with Aβ_25–35_ (20 μM). Aβ, β-amyloid; BDNF, brain-derived neurotrophic factor.

**Figure 6:**
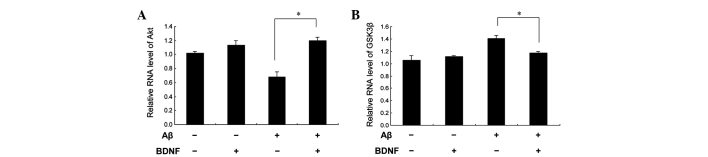
Levels of mRNA expression of (A) *Akt* and (B) *GSK3β*. mRNA expression was measured by reverse transcription quantitative polymerase chain reaction. Results are shown as the mean ± standard error of the mean of three independent experiments. ^*^P<0.05 vs. the group treated only with Aβ_25–35_ (20 μM). Aβ, β-amyloid; BDNF, brain-derived neurotrophic factor; *GSK3β*, glycogen synthase kinase-3β.

Akt is a known pro-survival kinase that is activated by phosphorylation via PI3K (13). The BDNF-mediated phosphorylation of Akt was measured, as shown in Fig. 5. The treatment of cells with a combination of Aβ_25–35_ (20 μM) and BDNF (10 ng/ml) induced a significant increase in the level of p-Akt compared to that in the cells treated only with Aβ_25–35_ (20 μM). This was supported by the measurement of the *Akt* mRNA levels, as shown in Fig. 6A.

### GSK3β modulation of BDNF-mediated signaling

**Figure 7:**
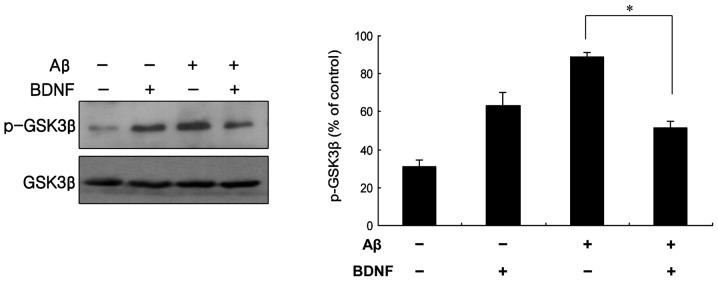
BDNF-mediated activation of GSK3β in SH-SY5Y neuroblastoma cells. Representative western blots showing the protein levels of phosphorylated (p-GSK3β) and total GKS3β in SH-SY5Y cells. The western blots presented represent three independent experiments with similar results. ^*^P<0.05 vs. the group treated with only Aβ_25–35_ (20 μM). Aβ, β-amyloid; BDNF, brain-derived neurotrophic factor; GSK3β, glycogen synthase kinase-3β.

GSK3β is known to be inhibited by serine phosphorylation mediated by Akt, which in turn is induced by growth factor signaling (11). Treatment of cells with a combination of Aβ_25–35_ (20 μM) and BDNF (10 ng/ml) induced a significant decrease in the level of p-GSK3β, compared with cells treated only with 20 μM Aβ_25–35_ (Fig. 7). The mRNA levels obtained for *GSK3β* supported this finding (Fig. 6B). These data indicate that GSK3β contributed to the neuroprotective effect of BDNF against the neurotoxicity of Aβ_25–35_.

## Discussion

Aβ is a 40–42 amino acid peptide fragment derived by proteolysis from the integral membrane protein known as Aβ precursor protein (14). Aβ, the central constituent of senile plaques in AD, is known to exert toxic effects on neurons (15). Aβ_25–35_ is the shorter toxic fragment corresponding to amino acids 25–35, which encompasses the β sheet of the full protein (16). Therefore, Aβ_25–35_ has been used to assess toxicity in the AD *in vitro* models (10). However, the mechanisms by which the Aβ peptide exerts its neurotoxic effect are not yet understood.

BDNF levels have been observed to decrease in the parietal cortex and hippocampus of patients with AD (17). BDNF serum concentrations have been reported to correlate with the severity of dementia (18). Furthermore, in AD brains, neurons containing neurofibrillary tangles do not contain BDNF-immunoreactive material, whereas neurons that stain intensely for BDNF are devoid of tangles (19).

In the present study, the protective effect of BDNF against toxicity induced by Aβ_25–35_ in SH-SY5Y cells was assessed. The results indicated that exposure of SH-SY5Y cells to Aβ_25–35_ caused a decrease in viability and the cell apoptosis rate increased, while the expression of p-Akt decreased and p-GSK3β increased. In addition, p-Akt and p-GSK3β may be associated with the Aβ_25–35_-induced cell apoptosis.

The PI3K/Akt pathway is important for cell survival. PI3K enhances neuroprotection by regulating the level of phosphorylation and activation of Akt. Akt activity can be modulated by phosphorylation of either Thr308 or Ser473 (20). Phosphorylation of Thr308 has been reported to be stimulated by dopamine receptor activation, while phosphorylation of Ser473 is regulated by the activation of the N-methyl-D-aspartate receptor (21–23). Therefore, phosphorylation and activation of Akt may underlie the observed protective effect of BDNF.

GSK3β is a substrate of Akt, which phosphorylates and inhibits GSK3β. Furthermore, GSK3β is subject to inhibitory regulation by growth factors that activate the PI3K/Akt pathway (24). Beaulieu *et al* (25) reported that activation of the PI3K/Akt pathway can increase the phosphorylation of GSK3β, thereby inhibiting the activity of the latter; thus, Akt may be a regulator of GSK3β. The present study indicated that the neuroprotective effect of BDNF against Aβ_25–35_ toxicity was mediated by the inhibitory effect of Akt on GSK3β. In conclusion, the data showed that administration of BDNF exerts neuroprotective actions against the toxic effect of the Aβ_25–35_-induced apoptosis in SH-SY5Y cells, which involved PI3K/Akt activation and GSK3β phosphorylation. The mechanism and signaling pathways underlying neuronal degeneration induced by the Aβ peptide remain to be further elucidated.
